# An Efficient Deep Learning-Based High-Definition Image Compressed Sensing Framework for Large-Scene Construction Site Monitoring

**DOI:** 10.3390/s23052563

**Published:** 2023-02-25

**Authors:** Tuocheng Zeng, Jiajun Wang, Xiaoling Wang, Yunuo Zhang, Bingyu Ren

**Affiliations:** State Key Laboratory of Hydraulic Engineering Simulation and Safety, Tianjin University, Tianjin 300072, China

**Keywords:** large-scene construction sites, high-definition, images compressed sensing, EHDCS-Net, downsampling and pixelshuffle

## Abstract

High-definition images covering entire large-scene construction sites are increasingly used for monitoring management. However, the transmission of high-definition images is a huge challenge for construction sites with harsh network conditions and scarce computing resources. Thus, an effective compressed sensing and reconstruction method for high-definition monitoring images is urgently needed. Although current deep learning-based image compressed sensing methods exhibit superior performance in recovering images from a reduced number of measurements, they still face difficulties in achieving efficient and accurate high-definition image compressed sensing with less memory usage and computational cost at large-scene construction sites. This paper investigated an efficient deep learning-based high-definition image compressed sensing framework (EHDCS-Net) for large-scene construction site monitoring, which consists of four parts, namely the sampling, initial recovery, deep recovery body, and recovery head subnets. This framework was exquisitely designed by rational organization of the convolutional, downsampling, and pixelshuffle layers based on the procedures of block-based compressed sensing. To effectively reduce memory occupation and computational cost, the framework utilized nonlinear transformations on downscaled feature maps in reconstructing images. Moreover, the efficient channel attention (ECA) module was introduced to further increase the nonlinear reconstruction capability on downscaled feature maps. The framework was tested on large-scene monitoring images from a real hydraulic engineering megaproject. Extensive experiments showed that the proposed EHDCS-Net framework not only used less memory and floating point operations (FLOPs), but it also achieved better reconstruction accuracy with faster recovery speed than other state-of-the-art deep learning-based image compressed sensing methods.

## 1. Introduction

In recent years, high-definition images are being used more and more extensively to monitor large-scene construction sites [1,2,3,4,5,6,7]. High-definition monitoring images contain substantial pixel information, which introduces strains on efficient transmission and communication due to the limited communication bandwidth and computing resources, especially at some high-altitude civil engineering or hydraulic engineering sites in high mountain valleys where network conditions are usually harsh and computing resources are scarce [8,9,10]. Meanwhile, in terms of information theory, the higher the resolution of the image, the more redundancies it contains, and the greater the potential for compression and reconstruction [11]. Thus, developing an efficient and accurate image compression and reconstruction algorithm suitable for on-site applications is significantly and urgently needed.

Compared to classic image compression standards, such as JPEG and JPEG2000 schemes, the emerging image compressed sensing techniques have better robustness, reconstruction quality, and higher computational efficiency [12,13,14], which makes them well-adapted for monitoring large construction sites in high mountain valleys with insufficient and unstable network bandwidth resources and computational resources. According to the Nyquist sampling theory, compared to classic image acquisition systems that have to gather samples at a sampling rate no less than twice the signal bandwidth and then compress the image, compressive sensing (CS) can directly capture compressed images at sampling rates below the Nyquist standard [11,15,16]. Additionally, the CS theory depicts that an image can be recovered with a small number of measurements using an appropriate optimization algorithm by exploiting the sparse characteristics of the signal in some transform domain. Specifically, assuming that $x\in R^{N}$ is a real-value signal that has sparse representation in some transform domain (such as discrete cosine transform (DCT) or wavelet), the CS theory states that it can be captured by taking the linearized CS measurements as follows:

(1) y=Φx

where $\Phi \in R^{M\times N}$ is a sampling matrix with M≪N and $y\in R^{M}$ is the CS measurement. The sampling rate, namely the ratio of M/N, is also called the measurement rate or CS ratio [11,16,17,18]. Image recovery from CS measurements requires solving an underdetermined linear inverse system, which can be expressed as

(2) $$\underset{x}{\min}ℜ\left(x\right),\;s.\;t.\;y=\Phi x$$

where $ℜ\left(x\right)$ is the regularization term. There have been a large number of studies proposing different strategies for solving this optimization problem. Among them, nonlinear iterative algorithms were early model-based traditional solutions, including sparse Bayesian learning, orthogonal matching pursuit (OMP), fast iterative shrinkage-thresholding algorithm (FISTA), approximate message passing (AMP), etc. [17,19,20,21,22]. Nevertheless, these methods have significant computational cost and poor reconstruction speeds [16,17].

Recently, with the rapid development of deep learning in the field of image processing, many deep learning-based CS methods have emerged [16,17,18,23,24,25,26,27,28]. They have been demonstrated to have outstanding performance by evaluating open-source datasets such as BSDS500, Set11, and BSD68 in research studies. Table 1 enumerates the performance of different image CS methods on Set11, where the peak signal-to-noise ratio (PSNR) and structural similarity index measure (SSIM) are largely the better metrics for image reconstruction quality. Among them, denoising-based approximate message passing (D-AMP) and deep compressed sensing (DCS) are traditional image CS methods, and their performance is not comparable to other deep learning-based CS methods. Among the state-of-the-art deep learning-based CS methods, CSNet^+^ builds an end-to-end block-based compressed sensing network using convolution layers to simulate the three procedures of block-based compressed sensing (BCS) (i.e., sampling, linear initial reconstruction, and nonlinear deep reconstruction subnetworks), which offers a good balance between reconstruction quality and speed since it has a relatively simple and efficient network architecture [16]. As shown in Table 1, although ReconNet runs slightly faster than CSNet^+^, its image reconstruction quality is not as good as CSNet^+^ at most CS ratios. AMP-Net is a little better than CSNet^+^ in terms of image reconstruction quality, but there is a difference of nearly 4 times in terms of computation speed; the running time is further lengthened when processing high-definition monitoring images of large-scene construction sites, which is a critical performance indicator. Additionally, based on such a network architecture design, CSNet^+^ achieves an adaptively learned sampling matrix and avoids blocking artifacts by effectively utilizing interblock information [16]. However, these deep learning-based image CS methods still face challenges in processing high-definition monitoring images of large-scene construction sites, namely the significant increase in computation and memory usage caused by the large number of convolution layers implemented in calculating the original image size in the deep network. This issue is not easily exposed in the CS community, since most common open-source datasets do not have very large image sizes (e.g., BSDS500, BSD68, BSD100, Set11, and Set14). However, this difficulty must inevitably be considered in the practical application of large-scene construction site monitoring because high-resolution images with nearly 2k resolution or more are usually monitored. In addition, these deep learning-based CS methods have not been applied and evaluated in construction site monitoring images. Take CSNet^+^ as an example, where the nonlinear transform subnetwork is composed of multiple stacked convolution layers. It is known that the computational effort and memory of one convolution layer are closely related to the resolution of the image, which can be expressed as:

(3) $$FLOPs=2\times C_{i}\times k^{2}\times C_{o}\times W\times H\;(includes\;bias)$$

(4) $$Memory=C_{o}\times \left(k^{2}\times C_{i}+1\right)+C_{o}\times W\times H\;(includes\;bias)$$

where *FLOPs* (floating point operations) measures the computations of one convolution layer; Memory consists of two parts, i.e., memory for the model (i.e., the first term in Equation (4)) and memory for the layer outputs (i.e., the second term in Equation (4)); $C_{i}$ represents the input channels; $C_{o}$ represents the output channels; k is the size of the square convolution kernel; and W, H are the width and height of the feature maps, respectively. Therefore, since state-of-the-art deep learning-based CS methods mainly perform convolutions on the original image size, ensuring the efficiency and accuracy of high-resolution monitoring image reconstruction remains challenging while using less memory occupation and computational cost, according to Equations (3) and (4). 

To address the above issues, this study presents an efficient deep learning-based high-definition image compressed sensing framework for large-scene construction site monitoring, dubbed EHDCS-Net, which draws on the simple and efficient network architecture of CSNet^+^ and is exquisitely designed by the rational organization of the convolutional, downsampling, and pixelshuffle layers, based on the three procedures of block-based compressed sensing. The EHDCS-Net framework consists of four parts, including the sampling, initial recovery, deep recovery body, and recovery head subnets. In terms of network structure functionality compared to CSNet^+^, the first two correspond to the sampling subnetwork and the linear initial reconstruction subnetwork of CSNet^+^, respectively, while the latter two correspond to the nonlinear deep reconstruction subnetwork of CSNet^+^. However, EHDCS-Net uses a pixelshuffle layer in the initial recovery subnet instead of the reshape and concatenation operations in CSNet^+^ for linear initial reconstruction, which has proven to be competitive in reconstructing images in the image super-resolution domain [18,29,30,31,32]. Meanwhile, different from CSNet^+^, which performs deep nonlinear transformations on the original image size, EHDCS-Net introduces a downsampling layer in the deep recovery body to downscale the feature map size before performing a nonlinear transformation, which effectively reduces memory occupation and computational cost in reconstructing images. After finishing the nonlinear transformation, a pixelshuffle layer is used again to recover the original image size and output the reconstruction residual, and then the combined reconstructed image generated from a skip connection between the residual and initial reconstructed image is fed to the recovery head for a finer restoration. To increase the nonlinear reconstruction capability on the downscaled feature maps, the ECA attention mechanism is further integrated into the deep recovery subnet. In addition, EHDCS-Net employs $l_{1}$ loss rather than $l_{2}$ loss, which is widely used in deep learning-based image CS methods (e.g., CSNet^+^, AMP-Net, and ISTA-Net), and comparison experiments were conducted to verify the superiority of $l_{1}$ loss in this framework. The framework was tested on large-scene monitoring images from a real hydraulic engineering megaproject and extensive comparative experiments were performed to illustrate that the proposed EHDCS-Net framework not only exhibited less memory usage and FLOPs, but it also achieved better reconstruction accuracy with faster recovery speed than other state-of-the-art deep learning-based image CS methods.

The remainder of this paper is organized as follows: Section 2 provides an overview of related work; Section 3 presents the methodology; Section 4 provides the analysis and comparisons of the experimental results; and Section 5 presents the conclusions. 

## 2. Related Work

Deep learning has demonstrated its superiority in various image processing problems (i.e., image enhancement [33,34], image super-resolution [35,36], and image classification [37,38]). In recent years, deep learning-based CS methods also have been shown to significantly outperform traditional model-based methods (e.g., discrete wavelet transform (DWT), total variation augmented Lagrangian alternating-direction algorithm (TVAL3), and D-AMP) in image compressed sensing [11,16,17,18,23,24,25,26,27,28,39,40,41,42,43,44]. Existing deep learning-based CS methods can be mainly divided into block-by-block reconstruction methods [17,18,26,27,41,45] and end-to-end reconstruction methods [11,16,24,25,28]. Mousavi et al. [45] applied a stacked denoising autoencoder (SDA) to learn a structured representation from sampled data and computed a signal estimate in image compressed sensing. Kulkarni et al. [26] proposed a CNN, namely ReconNet, for image block intermediate reconstruction and an off-the-shelf denoiser for deblocking to obtain the final reconstructed image. Zhang and Ghanem [27] developed a strategy to solve the proximal mapping associated with the sparsity-inducing regularizer by nonlinear transforms, casting the iterative shrinkage-thresholding algorithm into ISTA-Net. Zhang et al. [18] presented a constrained optimization framework for adaptive sampling and the recovery of image CS, called OPINE-Net, which was composed of three subnets, including the sampling, initialization, and recovery subnets. Xu et al. [41] introduced a Laplacian pyramid reconstructive adversarial network (LAPRAN) that simultaneously produced hierarchies of reconstructed images with incremental resolution. Zhang et al. [17] designed AMP-Net by unfolding the iterative denoising process of the approximate message passing algorithm onto deep networks, which consisted of a sampling model for the block-by-block measurement of images and a reconstruction model for the iterative denoising process. Since block-by-block reconstruction methods will cause blocking artifacts, these methods need to consider this further to improve the quality of the reconstruction image [25]. For example, in AMP-Net [17], a deblocking module is integrated following the denoising module in the reconstruction model to eliminate blocking artifacts. In addition, an enhanced multiblock version of OPINE-Net, dubbed OPINE-Net^+^, was further developed to independently sample image blocks and jointly reconstruct them by exploiting the interblock relationship [18]. Therefore, compared to these methods, end-to-end reconstruction methods have the natural advantage of avoiding blocking artifacts by directly learning end-to-end mapping between measurements and the whole reconstructed images [25]. Sun et al. [11] proposed a subpixel convolutional generative adversarial network for the image reconstruction process, dubbed SCGAN, including a generator that learned the explicit mapping from the low-dimensional measurement to the high-dimensional reconstruction and a discriminator that learned the inherent image distribution by implementing adversarial training with the generator. Shi et al. [24] investigated CSNet to establish end-to-end mapping between the compressed samples and the reconstructed images by stacking convolutional layers following the traditional block compressed sensing smooth projected Landweber algorithm. The sampling subnetwork in CSNet consists of a convolution layer, which shows simplicity and effectiveness by avoiding complex artificial designs and adaptively learning the sampling matrix. The initial reconstruction in CSNet consists of convolution and combination layers for imitating the minimum mean square error linear estimation in traditional block-based compressed sensing (BCS) reconstruction. The deep reconstruction in CSNet consists of five convolution layers and the corresponding ReLU activation functions for implementing the nonlinear signal reconstruction process. CSNet has good performance in terms of reconstruction quality and speed; however, the nonlinear reconstruction capability achieved by simply stacking five convolutional layers is still slightly insufficient. In ref. [16], Shi et al. proposed CSNet^+^, further based on CSNet with reference to ResNet [46], using a residual learning structure to improve the deep reconstruction subnetwork, which achieved better reconstruction quality. However, the deep reconstruction subnetwork in CSNet^+^ still had the potential to exploit the attention mechanisms developed in residual learning in order to further improve the image representation capability and reconstruction quality. 

In summary, since most of the convolution calculations of these existing deep learning-based image CS methods are performed on the original image size to obtain the reconstructed image, there remain some challenges that need to be addressed for large-scene construction site monitoring. On the one hand, since the resolution of the images in most open-source datasets is relatively much smaller than the high-definition images of large-scene construction site monitoring (nearly 2k resolution or more), these methods have not yet been demonstrated to be effective for construction site monitoring images. On the other hand, since the higher resolution of the recovered image leads to more computation and memory consumption during the convolution calculation, it is challenging to recover these images with high quality and fast speed in cases that require as little computational effort and memory usage as possible. 

## 3. Methodology

**Figure 1:**
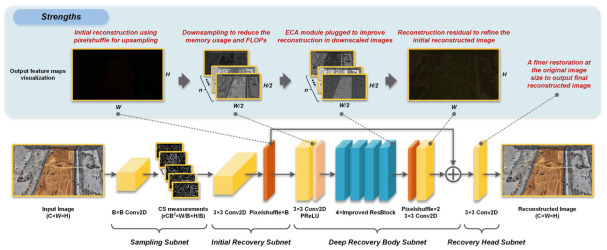
EHDCS-Net framework.

Figure 1 presents the proposed EHDCS-Net framework. Since CSNet^+^ has a simple and distinct end-to-end architecture based on the three operations of BCS and exhibits competitive performance in deep learning-based image CS methods, as discussed in Section 1, the EHDCS-Net framework learns from the CSNet^+^ architecture, which is also based on the three procedures of BCS and consists of four parts: the sampling, initial recovery, deep recovery body, and recovery head subnets. The sampling subnet is the same as that in CSNet^+^, which maintains the ability to adaptively learn the sampling matrix as an encoder to generate CS measurements. The initial recovery subnet is used to recover the initial reconstructed image from the CS measurements, in which we introduce the pixelshuffle layer for efficient and accurate linear reconstruction in order to replace the combination layer that comprises the reshape and concatenation operations in CSNet^+^. The pixelshuffle layer is widely used in the field of image super-resolution and has been proven to have remarkable upsampling capabilities in reconstructing images [18,29,30,31,32]. The deep recovery body is designed to implement deep nonlinear transformations on downscaled feature maps, and a downsampling layer is first introduced to save memory usage and FLOPs. In the main part of the deep recovery body, there are four stacked improved ResBlocks that integrate the ECA attention mechanism to further improve the nonlinear reconstruction at downscaled feature maps. At the end of the deep recovery body, the downscaled feature maps are restored to the original image size by a pixelshuffle layer, and then the reconstruction residuals obtained from the deep nonlinear transformation and the initial reconstruction image are added by a skip connection. Finally, the recovery head subnet is devised as a finer restoration of the original image’s size to further improve the quality of the final image reconstruction. The initial recovery, deep recovery body, and recovery head together form a decoder to efficiently and accurately recover the image with less memory occupation and computational cost. 

### 3.1. Sampling Subnet

Assume the image size is C × W × H, where C represents the channels of the image, W is the width of the image, and H is the height of the image. The sampling subnet is a convolution layer for sampling the image into feature maps of size rCB^2^ × W/B × H/B. The CS measurements consist of each nonoverlapping block sampling, where r represents the sampling ratio and B is the convolution kernel size. This is constructed by converting Equation (1) to a convolution calculation. Based on the BCS theory, each block is denoted by $x_{i}$ with a size of C × B × B, of which the CS measurement is expressed by $y_{i}=\Phi_{B}x_{i}$, where *i* represents the *i*th block and $\Phi_{B}$ is the sampling matrix of size rCB^2^ × CB^2^. $\Phi_{B}$ is similar to the rCB^2^ convolutional filters of size C × B × B with a stride of B × B, corresponding to a convolution layer for conducting nonoverlapping sampling. Notably, there is no bias in this convolution layer, and no activation function follows this layer [16]. This design of the sampling subnet, which inherits the advantages of CSNet/CSNet^+^, ensures that the sampling matrix can be adaptively learned by jointly training this convolution layer and the recovery network [16,24]. For large-scene construction site monitoring images, C is 3, W is 1920, and H is 1080, and we set B as 30, which can divide W and H and is close to the setting in most BCS method experiments (i.e., 32 or 33) in the CS community. Therefore, if r is 0.1, then there are 270 filters in this convolution layer.

### 3.2. Initial Recovery Subnet Using Pixelshuffle

**Figure 2:**
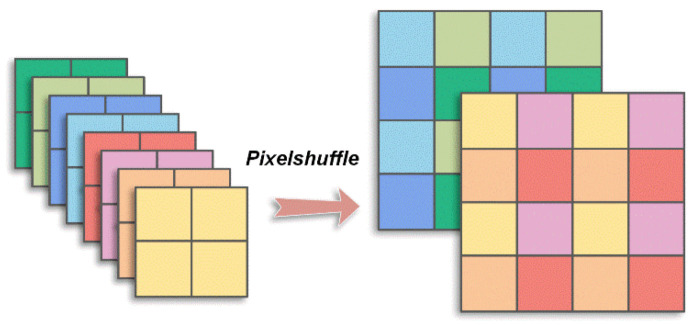
Pixelshuffle illustration.

The initial recovery subnet is composed of two sequential layers, namely the convolution and pixelshuffle layers, which mimic the process of generating the initial reconstructed image by utilizing a pseudoinverse matrix according to BCS. Given measurement $y_{i}$, the initial recovery result of each block $x_{i}$ can be computed by:

(5) $$x_{i}={\hat{\Phi }}_{B}^{}y_{i}$$

where ${\hat{\Phi }}_{B}^{}$ is a matrix of size *CB*^2^ × *rCB*^2^, which is adaptively optimized in training. Similarly, a convolution layer with *CB*^2^ filters of size *rCB*^2^ × 1 × 1 is constructed to obtain $x_{i}$, which is practically a tensor of size *CB*^2^ × 1 × 1 and corresponds to an image block of size *C* × *B* × *B*. There is also no bias, and the stride is set as 1 × 1 in this convolution layer. In CSNet^+^, a combination layer is simply used to reshape and concatenate all the reconstructed vectors, $x_{i}$, following the convolution layer to obtain the initial reconstructed image [16,24]. In the EHDCS-Net framework, we utilize a pixelshuffle layer to replace the combination layer, which has demonstrated good performance in many image super-resolution applications [29,30,31,32]. The pixelshuffle layer reshapes each tensor *CB*^2^ × 1 × 1 into tensor *C* × *B* × *B* and forms the initial reconstructed image. Figure 2 clearly illustrates the pixelshuffle layer. The initial recovery subnet absorbs the properties of the initial reconstruction part of CSNet^+^ that take full advantage of the intra- and interblock information of the image.

### 3.3. Deep Recovery Body Subnet Using Downsampling, Pixelshuffle, and ECA Attention Mechanism

The major deep learning-based image CS methods perform convolutions on the original image size during reconstruction, which has a significant impact on the memory allocation and FLOPs according to Equations (3) and (4); thus, in the EHDCS-Net framework, we designed a downscaled block to reduce the size of the pixelshuffle layer output at the beginning of the deep recovery body. The downscaled block consists of a convolutional downsampling layer and an activation layer, which is expressed as operation $D\left({\hat{x}}^{op}\right)$:

(6) $$D\left({\hat{x}}^{op}\right)=A(W_{ds}\circ {\hat{x}}^{op}+{ℬ}_{ds})$$

where ${\hat{x}}^{op}$ is the output of the pixelshuffle layer, namely the initial reconstructed image; $W_{ds}$ corresponds to n filters of size C × 3 × 3; $B_{ds}$ is the biases of size *n* × 3; ∘ represents the convolution with a stride of 2 × 2; and A(⋅) represents the activation function. In the experiment, *n* is set to 64, and A(⋅) is specified as the PReLU activation function, which has been shown to perform better than the commonly used ReLU activation function [47]. The outputs of the downscaled block are feature maps that have been reduced two-fold with respect to the length and width. These feature maps denote the high-dimensional features while reducing the computational load of the convolution in the deep recovery body and increasing computational efficiency. 

**Figure 3:**
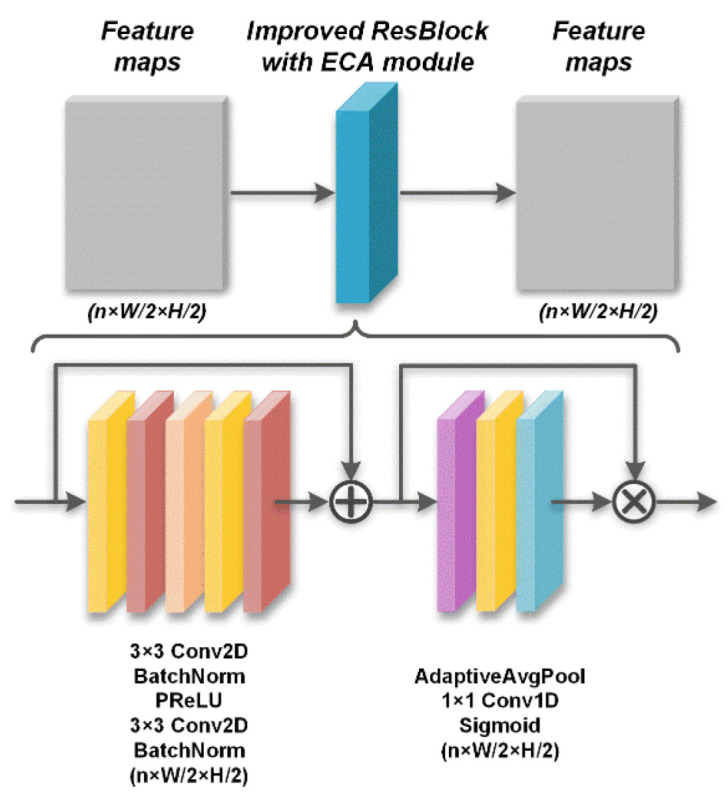
Improved ResBlock with ECA module framework.

After capturing the high-dimensional and downscaled feature maps of the initial reconstructed image, the deep recovery body employs the improved cascaded ResBlock with the attention mechanism of the efficient channel attention (ECA) module [48] added to the normal ResBlock. The ECA module is a channelwise attention mechanism that performs feature recalibration and improves the representational power when inserted as a module into a deep network [48]. The normal ResBlock is composed of a particular combination of these layers, including the convolution, batch normalization, and activation layers. The cascaded ResBlock is expressed as

(7) $$R^{i}=T\left(W_{r}^{i2}*A\left(T\left(W_{r}^{i1}*R^{i-1}+B_{r}^{i1}\right)\right)+B_{r}^{i2}\right)+R^{i-1}$$

where $T\left(\cdot \right)$ represents the batch normalization; $R^{i}$ is the output of the *i*th ResBlock, in which there is a short skip connection between the input and the output of the batch normalization layer; $W_{r}^{i1}$ and $B_{r}^{i1}$ correspond to the n filters of size *n* × 3 × 3 and biases of size *n* × 3, respectively, in the first convolution layer; $W_{r}^{i2}$ and $B_{r}^{i2}$ have the same sizes as $W_{r}^{i1}$ and $B_{r}^{i1}$, respectively, in the second convolution layer; ∗ represents the convolution with a stride of 1 × 1; and A(⋅) is a PReLU activation function. $R^{0}=D\left(\hat{x}\right)$. In the improved ResBlock, the ECA module inserted behind the normal ResBlock is depicted as operation $E\left(R\right)$:

(8) $$E\left(R\right)=A_{e}\left(W_{e}⊙P\left(R\right)\right)⊗R$$

where R is the output of the normal ResBlock; $P\left(\cdot \right)$ represents the adaptive average pooling; $W_{e}$ corresponds to one filter of size 1 × 3; ⊙ represents 1D convolution with a stride of 1 and a padding of 1; ⊗ denotes elementwise multiplication; and $A_{e}(\cdot )$ is a sigmoid activation function. With the insertion of the ECA module, the improved cascaded ResBlock can be expressed as follows by integrating Equations (7) and (8):

(9) $$\begin{array}{c} R_{ECA}^{i}=A_{e}\left(W_{e}^{i}⊙P\left(T\left(W_{r}^{i2}*A\left(T\left(W_{r}^{i1}*R_{ECA}^{i-1}+B_{r}^{i1}\right)\right)+B_{r}^{i2}\right)+R_{ECA}^{i-1}\right)\right)\\ \;⊗T\left(W_{r}^{i2}*A\left(T\left(W_{r}^{i1}*R_{ECA}^{i-1}+B_{r}^{i1}\right)\right)+B_{r}^{i2}\right)+R_{ECA}^{i-1} \end{array}$$

where $R_{\mathrm{ECA}}^{i}$ is the output of the *i*th improved ResBlock, $i\in \left\{1,2,3,4\right\}$. In the EHDCS-Net framework, the amount of improved cascaded ResBlock in the deep recovery body is set to 4. $R_{\mathrm{ECA}}^{0}=D\left(\hat{x}\right)$. Figure 3 shows the specific structure and corresponding network layers of the improved ResBlock with the ECA module.

To restore the feature maps to the original image size, the upsampling block is performed after the nonlinear signal reconstruction implemented by the improved cascaded ResBlock on the downscaled feature maps. The upsampling block consists of two layers, the pixelshuffle and convolution layers, which can be expressed as operation $U\left(R_{ECA}\right)$:

(10) $$U\left(R_{ECA}\right)=W_{u}*S\left(R_{ECA}\right)+B_{u}$$

where $R_{\mathrm{ECA}}$ is the output of the last improved cascaded ResBlock; $S\left(\cdot \right)$ represents the pixelshuffle that reshapes feature maps of size $n\times \frac{W}{2}\times \frac{H}{2}$ into feature maps of size $\frac{n}{4}\times W\times H$; and $W_{u}$ and $B_{u}$ correspond to the C filters of size $\frac{n}{4}\times 3\times 3$ and biases of size $\frac{n}{4}\times 3$, respectively, in the convolution layer. Thus, the output of the upsampling block is feature maps with a size of C×W×H, which is the same size as the original image. As shown in Figure 1, the generated feature maps are the reconstruction residual, which can be considered supplementary information for refining the initial reconstructed image. Therefore, at the end of the deep recovery body, a long skip connection is added between the reconstruction residual $U\left(R_{ECA}\right)$ and the initial reconstructed image $\hat{x}$ to obtain the fused reconstructed image and accelerate network convergence.

### 3.4. Recovery Head and Loss Function

Since the output of the deep recovery body is restored to the size of the original input image, to further refine the value of all channels in the recovery image and increase the network representation capability in reconstructing images at the original input size, a convolutional layer is set after the deep recovery body, which is called the recovery head, to output the final reconstructed image. Thus, the final reconstructed image is:

(11) $$H\left(U\left(R_{ECA}\right)+{\hat{x}}^{op}\right)=W_{h}*\left(U\left(R_{ECA}\right)+{\hat{x}}^{op}\right)+B_{h}$$

where $W_{h}$ and $B_{h}$ correspond to *C* filters of size C×3×3 and biases of size C×3, respectively, in the convolution layer. 

The EHDCS-Net framework, which inherits the advantages of CSNet^+^, retains an end-to-end network, which means that given input image x, CS measurement y is captured by the sampling subnet, and recovery image $\tilde{x}$ is reconstructed by the initial recovery, deep recovery body, and recovery head subnets, in turn, from CS measurement y. Therefore, CS measurement y can be considered an intermediate variable, and to train the entire end-to-end EHDCS-Net, the loss function can be simplified to consider only the loss between input image x and the corresponding image of output reconstruction $\tilde{x}$. There are two common loss functions that measure this difference: mean square error (MSE) and mean absolute error (MAE). The MSE is also called $l_{2}$ loss, which is the most popular loss function used in deep learning-based image CS methods, and it is defined as:

(12) $$l_{MSE}=\frac{1}{N}\sum_{i=1}^{N}{\left\|x_{i}-{\tilde{x}}_{i}\right\|}_{2}^{2}$$

where *i* denotes the index of the image in the training set. However, there are some studies that experimentally point out that training with $l_{2}$ loss may not always be the best choice in different applications [29,32,49]. The other loss function MAE is also called $l_{1}$ loss, which is formulated as:

(13) $$l_{MAE}=\frac{1}{N}\sum_{i=1}^{N}{\left\|x_{i}-{\tilde{x}}_{i}\right\|}_{1}$$

There is a growing number of studies in image CS, image restoration, and super-resolution problems using this loss function [29,32,50,51,52]. In EHDCS-Net, $l_{1}$ loss was selected as the loss function and we experimentally verified that $l_{1}$ loss can improve the image reconstruction quality better than $l_{2}$ loss when training EHDCS-Net. Thus, given a training set ${\left\{x_{i},x_{i}\right\}}_{i}^{N}$, the loss function of EHDCS-Net can be expressed by:

(14) $$L\left(\Theta \right)=\frac{1}{N}\sum_{i=1}^{N}{\left\|H\left(U\left(R_{ECA}\right)+{\hat{x}}_{i}^{op}\right)-x_{i}\right\|}_{1}$$

where Θ represents the trainable parameters of EHDCS-Net, and ${\left\|\right\|}_{1}$ is the $l_{1}$ norm. It is worth noting that, similar to CSNet^+^, the sampling subnet and all recovery subnets of EHDCS-Net are jointly trained as a whole yet they can also be employed separately.

## 4. Discussion

The proposed EHDCS-Net framework was tested on large-scene construction site high-definition monitoring images collected from a real hydraulic engineering megaproject. In this section, numerous numerical experiments were performed to validate the effectiveness and efficiency of EHDCS-Net. In accordance with the common evaluation metrics of image CS, the reconstruction quality of the large-scene construction site high-definition monitoring images, reconstruction speed, and the corresponding computational resource consumption were considered to illustrate the superiority of EHDCS-Net over other state-of-the-art methods.

### 4.1. Training Details

**Figure 4:**
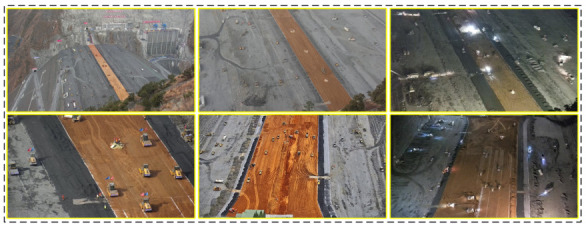
Some examples of large-scene construction site high-definition monitoring images.

A total of 4335 large-scene construction site high-definition monitoring images with 1920 × 1080 resolution were collected to compose the dataset. Among them, 400 images were used as the test set, 120 images were used as the validation set, and the remaining 3815 images were used for training. Figure 4 shows some examples of large-scene construction site high-definition monitoring images. Considering that different image CS methods require training images of different sizes, two training sets were generated based on the availability of trainable deblocking operations for different methods [17]: (a) training set 1 contained 36,000 subimages with a size of 99 × 99 that were randomly cropped from 3815 images of the training set [16]; (b) training set 2 contained 108,000 subimages with a size of 33 × 33 that were randomly cropped from 3815 images of the training set [27]. The validation set was applied to determine the best model for testing. For a fair comparison, the luminance components of the image were used as a comparison basis for calculating the evaluation metrics. Regarding image reconstruction quality, the peak signal-to-noise ratio (PSNR) and structural similarity index measure (SSIM) are the most commonly used metrics for evaluation [16,17,18,24,26,27]. For both indicators, higher values indicated better image reconstruction quality. Referring to other methods for selecting the optimal model, the model with the highest average PSNR value calculated on the validation set in each training epoch was chosen as the optimal model for testing [17].

In the training phase, the training epoch of EHDCS-Net was set to 100 and the batch size was 64. The learning rate was initialized to 0.0004 and decreased by half every 30 epochs. The optimizer was set as Adam for training, and the default settings were used for other hyperparameters of Adam [16]. A range of CS ratios *r* {1%, 4%, 10%, 25%, 50%} for training was used to analyze and compare the performance of the model under different CS ratios. Other network parameters were in accordance with the description in Section 3, i.e., *B* = 30, *C* = 3, and *n* = 64. The network was implemented based on the PyTorch framework. All experiments were performed on a workstation with a 64-bit Ubuntu 16.04 operating system with the following hardware configuration: a 45-core Intel Xeon(R) Gold 6132 CPU @ 2.60 GHz, 128 G RAM, and 2 × NVIDIA Quadro GV100.

**Figure 5:**
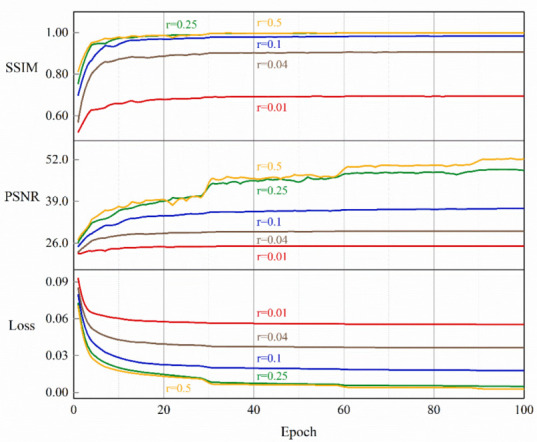
Training loss and validating PSNR and SSIM curves at different CS ratios.

As shown in Figure 5, the training process of EHDCS-Net with different CS ratios was portrayed by the curves of the SSIM of the validation set, the PSNR of the validation set, and training loss converging continuously with the training epochs. As the CS ratio increased, the curves of the PSNR and SSIM indicators also shifted upward, indicating that image reconstruction quality also improved. In addition, the improvement was more pronounced at lower CS ratios (i.e., *r* = 0.01, *r* = 0.04, and *r* = 0.1) and less pronounced at larger CS ratios (i.e., *r* = 0.25 and *r* = 0.5). Meanwhile, the upper boundary of the SSIM metric was 1, and the SSIM value was 0.9993 at a CS ratio of 0.5, which was quite close to 1, suggesting that CS image reconstruction at high CS ratios restored the original image quite well. 

### 4.2. Comparison of EHDCS-Net and CSNet^+^

**Figure 6:**
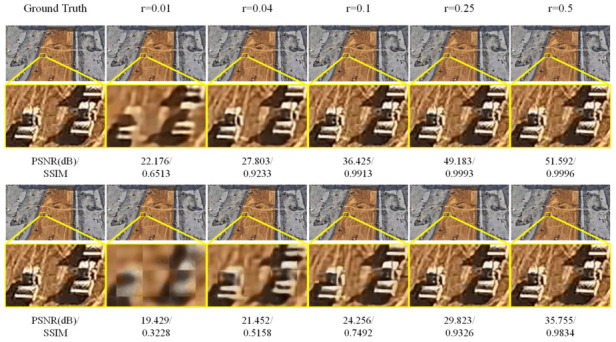
Visual quality comparison of large-scene construction site monitoring image CS recovery between CSNet^+^ and EHDCS-Net at different CS ratios. The first row is images reconstructed by EHDCS-Net and the second row is images reconstructed by CSNet^+^.

**Figure 7:**
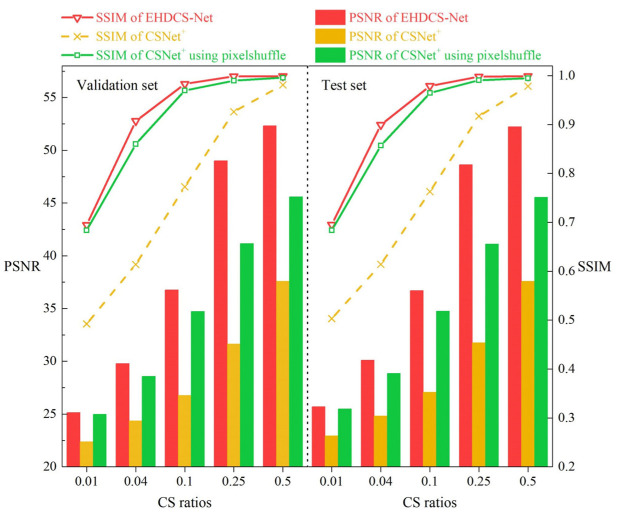
Comparisons of PSNR and SSIM values between CSNet+, CSNet+ using pixelshuffle, and EHDCS-Net on the validation and test sets, respectively, at different CS ratios.

Since both EHDCS-Net and CSNet^+^ are end-to-end network frameworks developed based on the three procedures of BCS, the images recovered by both methods at all CS ratios were first visualized to fully illustrate the superiority of EHDCS-Net compared to CSNet^+^ in the high-definition monitoring image reconstruction of large-scene construction sites. As shown in Figure 6, the same test image was reconstructed by EHDCS-Net and CSNet^+^ at different CS ratios. The first row shows the images recovered by EHDCS-Net, while the second row shows the images recovered by CSNet^+^. Each column represents the recovered image at the same CS ratio, except for the first column, which represents the corresponding ground truth image. For a clearer comparison, some details in the recovered image have been enlarged to directly visualize the reconstruction quality. The recovered image was captured at an altitude of 130 m from the large-scene construction site in order to be able to monitor the entire site, in which the rollers on the construction site can be more clearly identified via reconstruction of EHDCS-Net than with CSNet^+^. The PSNR and SSIM values are listed below each corresponding recovered image. Horizontally, the image reconstruction quality recovered by the same method improved with a higher CS ratio from the enlarged part. The details were shown more clearly and sharply. Vertically, the reconstructed result of EHDCS-Net was finer, smoother, clearer, and sharper than the result of CSNet^+^ at the same CS ratio. Meanwhile, the values of PSNR and SSIM also demonstrated the above statements. In terms of PSNR values, EHDCS-Net had a significant improvement over CSNet^+^. Figure 7 first shows a more comprehensive comparison of the PSNR and SSIM results regarding EHDCS-Net and CSNet^+^ on the validation and test sets, respectively. It can be seen that the PSNR and SSIM values of EHDCS-Net on both the validation and test sets were significantly superior to those of CSNet^+^ at all CS ratios. For PSNR, as the CS ratio increased, the improvement of EHDCS-Net increased as well; specifically, at *r* = 0.25, there was an enhancement of more than 17 and 16 dB on the validation and test sets, respectively. For SSIM, as the CS ratio increased the improvement of EHDCS-Net gradually decreased; specifically, at *r* = 0.04, there was a great increase of more than 0.29 and 0.28 on the validation and test sets, respectively. In addition, CSNet^+^ using pixelshuffle also showed significant improvement in the reconstruction quality of the large-scene construction site high-definition monitoring images, as shown in the comparison in Figure 7. Compared with CSNet^+^, CSNet^+^ using pixelshuffle achieved improvements in both PSNR and SSIM metrics, while EHDCS-Net further improved the deep recovery body subnet and recovery head as well as the loss function compared with CSNet^+^ using pixelshuffle, and thus had further gains in reconstruction quality. Overall, EHDCS-Net showed more effective and accurate based on the above results. 

**Figure 8:**
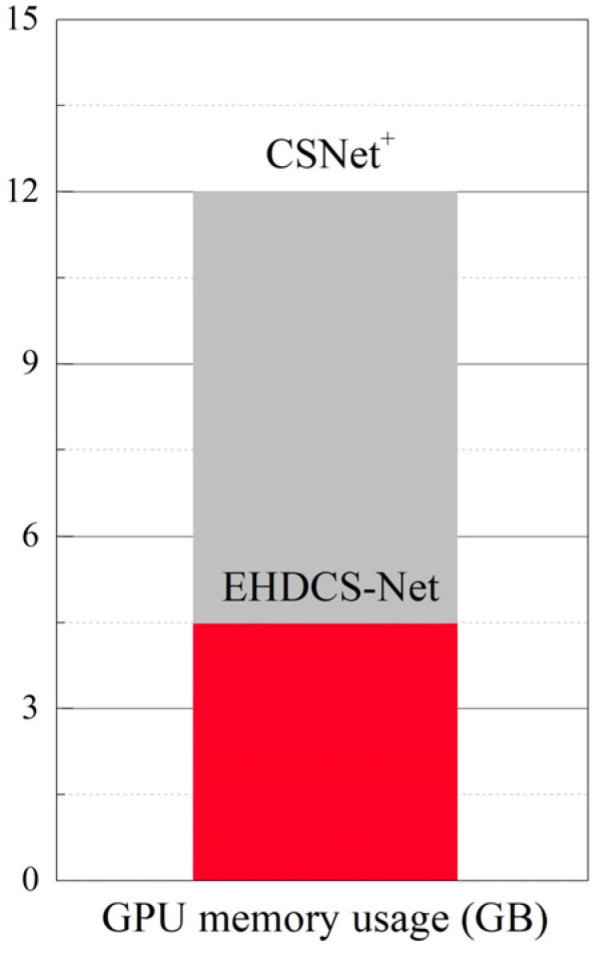
Comparison of GPU memory usage between CSNet^+^ and EHDCS-Net in the case of CS ratio = 0.1.

**Figure 9:**
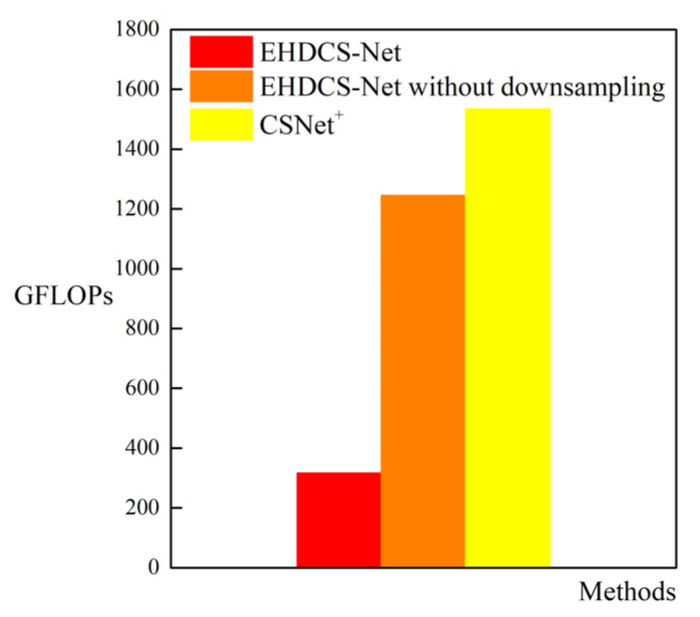
Comparison of GFLOPs among EHDCS-Net, EHDCS-Net without downsampling, and CSNet^+^ in the case of CS ratio = 0.1.

As shown in Figure 8, the comparison of GPU memory usage between EHDCS-Net and CSNet^+^ when recovering a large-scene construction site high-definition monitoring image is presented as a bar graph. EHDCS-Net considerably outperformed CSNet^+^ with GPU memory usage reduced by more than half. In addition, to better illustrate that the use of downsampling in EHDCS-Net sufficiently reduced the computational cost, EHDCS-Net was compared with EHDCS-Net without downsampling and CSNet^+^ for average FLOPs according to Equation (3) in recovering a large-scene construction site high-definition monitoring image at a CS ratio = 0.1, as shown in Figure 9. EHDCS-Net was significantly better than EHDCS-Net without downsampling and CSNet^+^, reducing the average FLOPs to about one-fourth of those with EHDCS-Net without downsampling and about one-fifth of those with CSNet^+^, respectively.

### 4.3. Validating the Performance of the Improved ResBlock and Different Loss Functions

In this subsection, since the ECA module was proven to be a promising and versatile lightweight attention mechanism, the performances of the improved ResBlock with and without the ECA module were first verified. Meanwhile, as described in Section 3.4, the capabilities of two common loss functions, $l_{1}$ loss and $l_{2}$ loss, were evaluated in training EHDCS-Net. In the above, the two methods used for comparison in all experiments had the same training settings in EHDCS-Net except for what was compared.

#### 4.3.1. Comparison with and without the ECA Module Attention Mechanism

**Figure 10:**
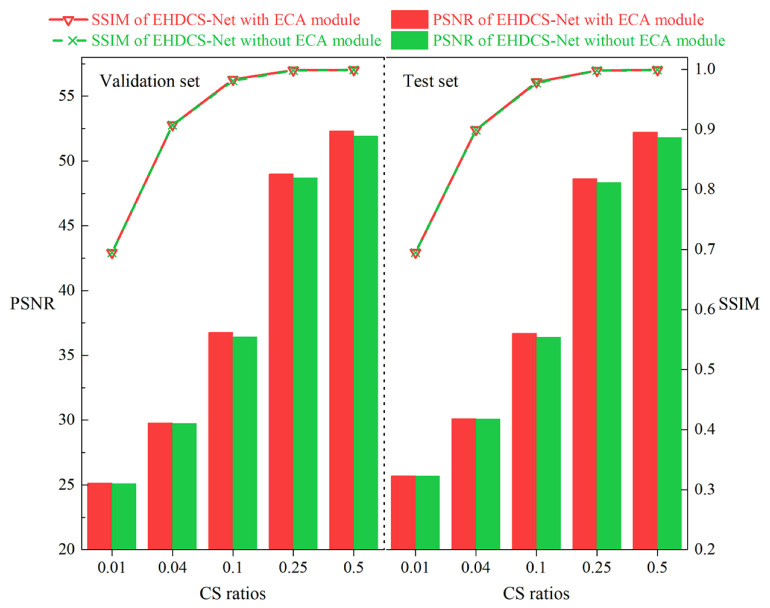
Comparisons of PSNR and SSIM values of EHDCS-Net with and without the ECA module on the validation and test sets, respectively, at different CS ratios.

As shown in Figure 10, the performances of EHDCS-Net with and without the ECA module plugged into the improved ResBlock were assessed on the validation and test sets, respectively, based on the PSNR and SSIM metrics. The comparison of PSNR values at different CS ratios between EHDCS-Net with and without the ECA module is presented as a bar graph, while the comparison of SSIM values at different CS ratios is displayed as a curve. In terms of the PSNR metric, the improvement increased with an increasing CS ratio; specifically, at *r* = 0.5, there was a major boost of 0.38 and 0.39 dB on the validation and test sets, respectively In terms of the SSIM metric, the improvement with the ECA module was not significant. Overall, the results of EHDCS-Net with the ECA module were slightly better than the results without the ECA module on both the validation and test sets.

#### 4.3.2. Comparison of $l_{1}$ Loss and $l_{2}$ Loss

**Figure 11:**
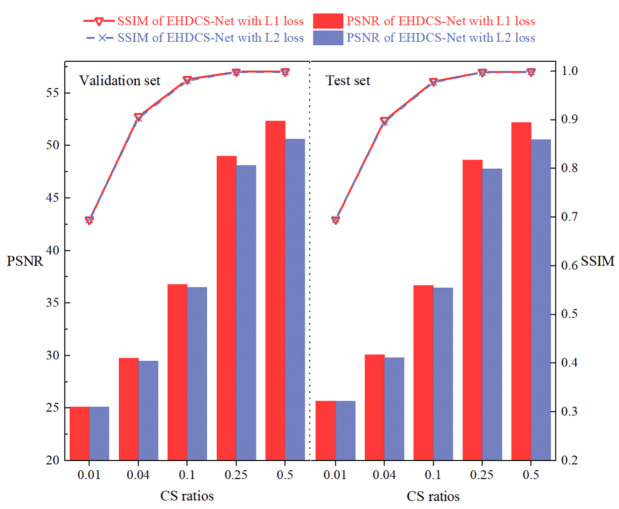
Comparisons of PSNR and SSIM values between EHDCS-Net using the $l_{1}$ loss and $l_{2}$ loss functions on the validation and test sets, respectively, at different CS ratios.

Figure 11 shows the performances of EHDCS-Net trained with $l_{1}$ loss and $l_{2}$ loss on the validation and test sets. It is evident from the PSNR metric that EHDCS-Net trained with $l_{1}$ loss was significantly better than that trained with $l_{2}$ loss. In addition, as the CS ratio increased, the PSNR values of EHDCS-Net trained with $l_{1}$ loss were increasingly better than those of EHDCS-Net trained with $l_{2}$ loss. In particular, at *r* = 0.5, the PSNR values of EHDCS-Net using the $l_{1}$ loss function were fully 1.72 and 1.66 dB higher than those using the $l_{2}$ loss function on the validation and test sets, respectively. On the other hand, the SSIM metric on both the validation and test sets exhibited little difference when using the $l_{1}$ or $l_{2}$ loss functions. Generally, the experimental results suggested using the $l_{1}$ loss function to train EHDCS-Net, verifying the conclusion about the loss function in Section 3.4.

### 4.4. Comparisons with State-of-the-Art Methods

**Figure 12:**
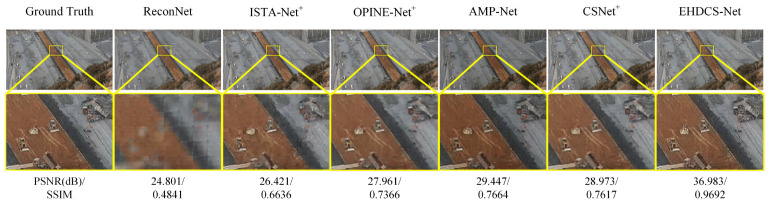
Visual quality comparison of large-scene construction site monitoring image CS recovery of different deep learning-based image CS methods in the case of CS ratio = 0.1.

In this subsection, EHDCS-Net was compared with four other state-of-the-art deep learning-based image CS methods, namely ISTA-Net^+^, OPINE-Net^+^, AMP-Net, and ReconNet. Since ISTA-Net^+^, OPINE-Net^+^, and CSNet^+^ were reported to perform relatively better than ISTA-Net, OPINE-Net, and CSNet in ref. [16,18,27], respectively, only the former was used here for comparison. Meanwhile, since AMP-Net also has many versions, the comparatively better-performing AMP-Net-9-BM was used as the AMP-Net involved in the comparison according to ref. [17]. The models engaged in the comparison were trained and tested according to the settings in their original papers. EHDCS-Net, CSNet^+^, OPINE-Net^+^, and AMP-Net were trained on training set 1, while ISTA-Net^+^ and ReconNet were trained on training set 2 because of their characteristics of recovering images in a direct block-by-block manner [17]. As shown in Figure 12, a large-scene construction site monitoring image reconstructed by these methods is visualized at a CS ratio of 0.1. From the enlarged part, it is obvious that EHDCS-Net restored more and finer details and sharper edges than the other methods, exhibiting superior reconstruction performance. Among them, both ReconNet and ISTA-Net^+^ reconstruction results revealed significant blocking artifacts since they are direct block-by-block reconstruction methods [16]. Additionally, EHDCS-Net outperformed all the other competing methods by a large margin in terms of PSNR and SSIM values. 

**Figure 13:**
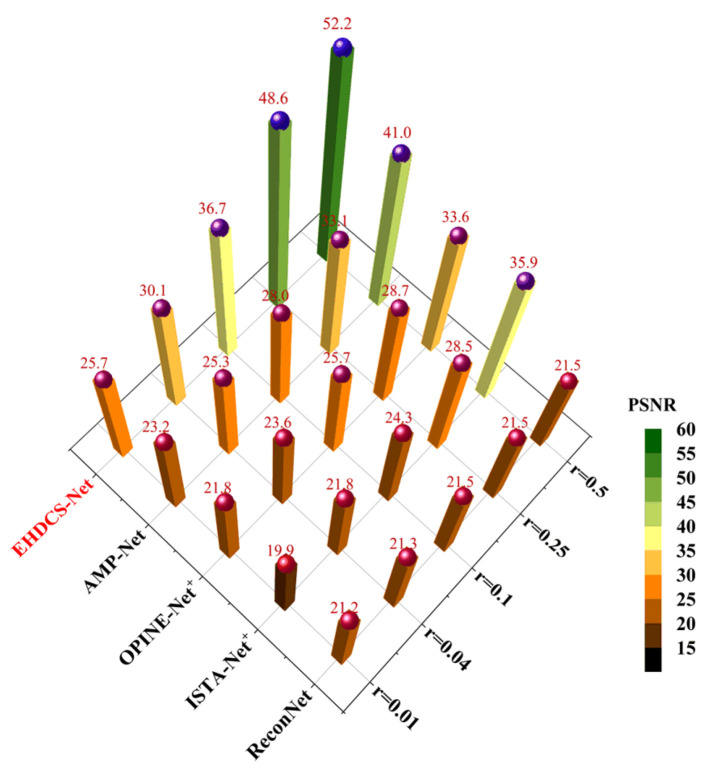
Comparison of average PSNR values of different deep learning-based image CS methods evaluated on the test set at different CS ratios.

**Figure 14:**
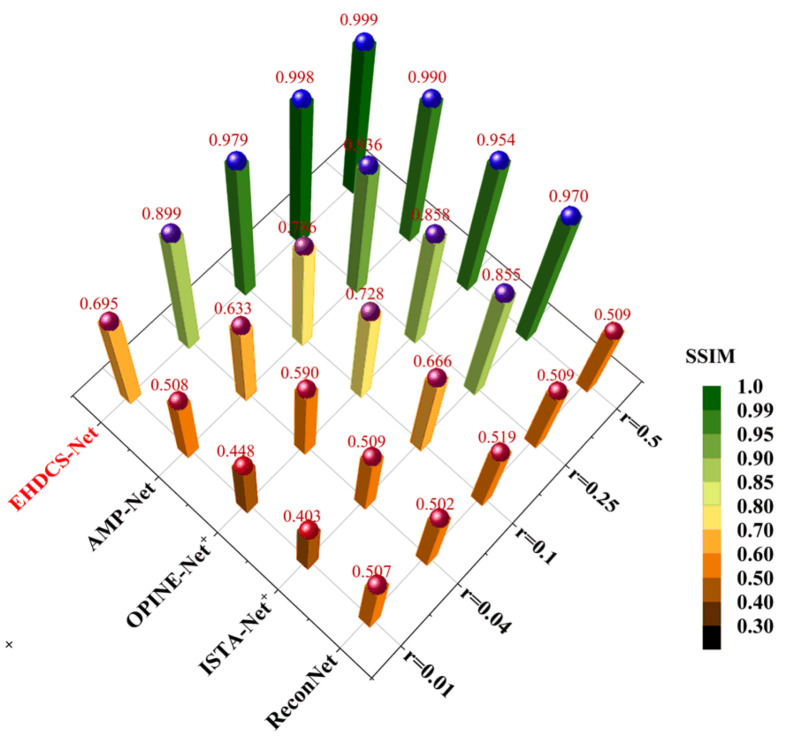
Comparison of average SSIM values of different deep learning-based image CS methods evaluated on the test set at different CS ratios.

Figure 13 shows a comparison of the average PSNR results of different deep learning-based image CS methods evaluated on the test set at different CS ratios. It is clear that EHDCS-Net achieved the best PSNR values at all CS ratios and is marked in red font in the figure. Compared with the best method of the other four deep learning-based CS methods, i.e., AMP-Net, EHDCS-Net improved the average PSNR values by more than 2.5, 4.85, 8.72, 15.52, and 11.24 dB with respect to CS ratios of 0.01, 0.04, 0.1, 0.25, and 0.5, respectively. Meanwhile, with increasing CS ratio, the superiority of EHDCS-Net became more significant. Figure 14 shows the comparison of the average SSIM results of different deep learning-based image CS methods evaluated on the test set at different CS ratios. Likewise, EHDCS-Net also achieved the highest average SSIM values on the test set at all CS ratios, and compared with the second-best method, AMP-Net, the average SSIM gains were more than 0.186, 0.266, 0.193, 0.062, and 0.009 with respect to CS ratios of 0.01, 0.04, 0.1, 0.25, and 0.5, respectively. With an increase in the CS ratio, the increment of SSIM decreased, which may have been a result of SSIM possessing an upper bound, while the other methods (e.g., AMP-Net) had already obtained quite high SSIM values in recovering the image at high CS ratios, thus there was relatively little room for increase. Therefore, this led to a more significant improvement of SSIM at a low CS ratio. In summary, all PSNR and SSIM values illustrated that EHDCS-Net exhibited the best image quality in recovering the large-scene construction site monitoring image. 

### 4.5. Running Time Comparisons

High-definition large-scene construction site monitoring images also stress the computational speed of image CS. Hence, the running time of the methods in reconstructing the high-definition images of large-scene construction sites is also an important performance metric to be considered. Table 2 provides a comparison of the average running time for different state-of-the-art deep learning-based image CS methods recovering a high-definition large-scene construction site monitoring image with a resolution of 1920 × 1080 in the case of CS ratio = 0.1. In the interest of a fair comparison, all methods were tested based on the PyTorch framework implementation. As Table 2 shows, although both EHDCS-Net and CSNet^+^ are end-to-end network frameworks developed based on the three procedures of BCS, the computational speed of EHDCS-Net was approximately 44 times faster than that of CSNet^+^, which fully demonstrated the efficiency and advantages of the EHDCS-Net framework in improving the reconstructed image quality while also increasing the computational speed. In addition, EHDCS-Net was also 4.6 times faster than the second fastest method (i.e., ISTA-Net^+^). Since other methods recovered the reconstructed image with the original image’s size, the results showed that EHDCS-Net had the fastest computational speed performance, which also clearly demonstrated the importance of performing nonlinear transformations on downscaled feature maps in reducing computations and increasing computational speed.

### 4.6. FLOPs and Memory Usage Comparisons

**Figure 15:**
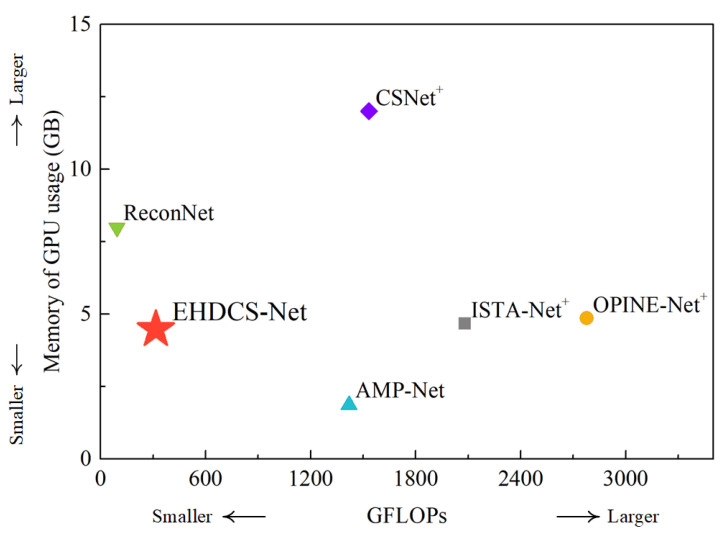
GFLOPs and GPU memory usage comparisons of different deep learning-based image CS methods for reconstructing a large-scene construction site monitoring image (1920 × 1080) in the case of CS ratio = 0.1.

According to Equations (3) and (4), the FLOPs and memory usage are not negligible when reconstructing large-scene construction site high-definition monitoring images. Figure 15 shows a comparison of the GFLOPs and GPU memory usage of different deep learning-based image CS methods in reconstructing large-scene construction site monitoring images with a resolution of 1920 × 1080 in the case of CS ratio = 0.1. In the figure, the method closer to the bottom left indicates less computational cost and GPU memory usage, which means that the method is more preferable. Compared with EHDCS-Net, ReconNet had fewer GFLOPs while exhibiting much larger GPU memory usage, and AMP-Net had less GPU memory usage while exhibiting many more GFLOPs. Therefore, EHDCS-Net was the best-performing method in balancing GFLOPs and GPU memory usage among all compared deep learning-based image CS methods, which also validated the effectiveness and efficiency of the fine design of the EHDCS-Net framework architecture in improving the reconstruction of large-scene construction site high-definition monitoring images. Meanwhile, considering that the high-definition monitoring image reconstruction quality of the other methods was not as impressive as the results of EHDCS-Net, referring to Figure 12, Figure 13 and Figure 14, the EHDCS-Net framework was the ideal combination of higher image reconstruction quality, lower computational costs, and less memory usage for large-scene construction site monitoring. 

## 5. Conclusions

To ensure the efficient transmission of high-definition monitoring images of large-scene construction sites with harsh network conditions and scarce computing resources, this study proposed an efficient deep learning-based high-definition image compressed sensing framework (EHDCS-Net) for large-scene construction site monitoring, which can achieve high-quality and fast end-to-end compressed sampling and reconstruction with low computational cost and memory consumption. The EHDCS-Net framework was developed based on the procedures of block-based compressed sensing, which consists of four parts: the sampling, initial recovery, deep recovery, and recovery head subnets. To accommodate the limited bandwidth and computing resources at construction sites, the framework utilizes nonlinear transformations on downscaled feature maps in reconstructing images, which in turn effectively reduces memory occupation and computational cost. Moreover, to further increase the nonlinear reconstruction capability on downscaled feature maps, the ECA attention mechanism was introduced to improve the performance of ResBlock in the deep recovery subnet. In addition, the $l_{1}$ loss function was used to train the EHDCS-Net instead of the widely used $l_{2}$ loss function, based on the experimental results of the comparison. This framework was tested on large-scene monitoring images from a real hydraulic engineering megaproject. A number of experiments illustrated that, compared to other state-of-the-art deep learning-based image CS methods, the EHDCS-Net framework had a more competitive performance with an ideal balance of better image reconstruction accuracy, faster recovery speed, lower computational cost, and memory usage at different CS ratios in recovering high-definition monitoring images of large-scene construction sites. Nevertheless, considering that the construction site may need to encrypt some specific confidential images, an encryption algorithm can be incorporated on the basis of this framework in subsequent research to ensure the security of high-definition monitoring image transmission at large construction sites.

## Figures and Tables

**Table 1:** Performance comparisons of different image CS algorithms on the Set11 dataset [17].

Algorithm	Metrics	CS Ratios				
		0.01	0.04	0.1	0.25	0.5
D-AMP	PSNR (dB)	5.58	11.28	19.87	31.62	37.34
	SSIM	0.0034	0.0971	0.3757	0.7233	0.8504
	Running time (s)	39.139 (CPU)				
DCS	PSNR (dB)	17.12	18.03	21.53	21.85	22.30
	SSIM	0.3251	0.2202	0.4546	0.5116	0.5452
	Running time (s)	0.036 (GPU)				
ReconNet	PSNR (dB)	20.16	24.29	27.63	32.07	37.42
	SSIM	0.5431	0.7382	0.8487	0.9246	0.9609
	Running time (s)	0.004 (GPU)				
ISTA-Net^+^	PSNR (dB)	17.48	21.14	25.93	32.27	38.08
	SSIM	0.4403	0.5947	0.7840	0.9167	0.9680
	Running time (s)	0.027 (GPU)				
CSNet^+^	PSNR (dB)	20.09	24.24	27.76	32.76	38.19
	SSIM	0.5334	0.7412	0.8573	0.9322	0.9739
	Running time (s)	0.007 (GPU)				
AMP-Net	PSNR (dB)	20.20	25.26	29.40	34.63	40.34
	SSIM	0.5581	0.7722	0.8779	0.9481	0.9807
	Running time (s)	0.027 (GPU)				

**Table 2:** Average running time (in seconds) of various deep learning-based image CS methods for reconstructing a large-scene construction site monitoring image (1920 × 1080) in the case of CS ratio = 0.1.

Methods	Average Running Time (s)
EHDCS-Net	0.0028
CSNet^+^	0.1236
AMP-Net	0.3253
ISTA-Net^+^	0.0129
OPINE-Net^+^	0.0151
ReconNet	0.0623

## Data Availability

Some data used of this study are available from the corresponding author, upon reasonable request.
